# Use of lead-210 as a novel tracer for lead (Pb) sources in plants

**DOI:** 10.1038/srep21707

**Published:** 2016-02-22

**Authors:** Handong Yang, Peter G. Appleby

**Affiliations:** 1 Environmental Change Research Centre, University College London, Pearson Building, Gower Street, London WC1E 6BT, UK; 2 Department of Mathematical Science, University of Liverpool, Liverpool L69 3BX, UK

## Abstract

Lead (Pb) released from anthropogenic sources and stored in environmental repositories can be a potential source for secondary pollution. Here we develop a novel approach for tracking Pb from atmospheric deposition and other sources in the environment using fallout ^210^Pb as a tracer, and apply the method to samples collected from Richmond Park, London, the UK. The mean ratio of ^210^Pb to total Pb in atmospheric depositions collected from a site adjacent to the park during August–October 2012 was 96 Bq mg^−1^, while the ratio in surface soils from the park was typically an order of magnitude lower. The difference between these values made it possible to trace the source of Pb in the plants. The ^210^Pb/Pb ratios in plants varied from 0 to 34 Bq mg^−1^ indicating different levels of Pb absorption from the atmosphere. The ratio in mosses had an average value of 22 Bq mg^−1^. This suggests that only around 20% of the Pb they contain was from direct atmospheric deposition, revealing possible limitations in the use of terrestrial mosses for monitoring atmospheric pollution. As well as tracking sources, variations in the ^210^Pb/Pb ratio can also reveal ways in which Pb is transferred within plants.

Over previous decades and centuries extensive anthropogenic emissions of environmentally persistent contaminants have substantially increased some trace metal concentrations in surface soils and sediments, and intensified the natural biogeochemical cycles of them in the biosphere over the past century^1^,^2^. For example, Pb pollution has been recorded by many natural archives such as lake sediments and peat bogs, and monitored by terrestrial mosses and other plants worldwide (cf 3,4,5,6).

The remobilisation of Pb from these repositories represents a significant potential source of secondary pollution. However, one of the main difficulties in studying this phenomenon is identifying the source of the Pb in a given context as the total Pb concentration in a given sample can include contributions from both natural and anthropogenic sources^7^,^8^,^9^,^10^. Further, the anthropogenic component can derive from a number of different processes such as coal burning, mining, smelting and car-exhaust emission (e.g.^7^,^11^,^12^). Since Pb from these different sources can have quite distinct isotopic signatures, data on the stable isotopic ratios (Pb has four stable isotopes ^204^Pb, ^206^Pb, ^207^Pb and ^208^Pb) can be used to yield information on its different geochemical origins. However, once anthropogenic and natural Pb are mixed in the environment, it is difficult to trace the pollutant’s movement.

Lead-210, an unstable (radioactive) isotope of Pb, has in the last few decades been widely used for dating environmental records in lake sediments and peat bogs^13^, for tracing soil erosion within a catchment, and assessing sediment distribution within a lake basin (cf^14^,^15^). Lead-210 has also been used for studying plant uptake of Pb (cf 2,16) though the methodology that is based on artificially spiking the soils with ^210^Pb and monitoring its subsequent concentrations in the plants and soils, rather than the isotopic ratio.

The ratio of the (atmospherically delivered) ^210^Pb to total Pb is normally considerably higher than that of the Pb in the environment repositories. The difference between these ratios in the environment makes it possible to trace the source of Pb into a recipient. Here we introduce a novel method for tracing the sources of Pb in the environment using the ratio of the atmospherically delivered ^210^Pb to total Pb. We exemplify the approach using samples collected from Richmond Park in London (Fig. 1) to assess the extent to which the different sources (direct atmospheric deposition, Pb stored in e.g. soils, dusts or tree trunks) affect Pb absorption and mobilization in plants.

## Theoretical Basis

The radionuclide ^210^Pb (half-life 22.26 y) occurs naturally as a member of the ^238^U decay series (Fig. 2). A fraction of the inert gas ^222^Rn, a product of ^226^Ra decay (half-life 1602 y) in soils, escapes to the atmosphere where it decays via a series of short-lived radionuclides to ^210^Pb. ^210^Pb atoms in the atmosphere are readily attached to airborne particles which are quickly removed to land surfaces and water bodies by wet and dry deposition. Fallout ^210^Pb accumulating in soils and sediments is called unsupported ^210^Pb, to distinguish it from the supported ^210^Pb that derives from *in situ* decay of the parent radionuclide ^226^Ra. The unsupported ^210^Pb will decay to near-zero concentrations over a period of around six ^210^Pb half-lives (~130 years). Supported ^210^Pb, which will usually be in radioactive equilibrium with ^226^Ra, is determined by measuring the ^226^Ra activity of the sample. Unsupported ^210^Pb is determined by subtracting the supported activity from the measured total ^210^Pb activity.

In order to examine the relationship between unsupported (or atmospherically delivered) ^210^Pb and total Pb we use the parameter *η* defined by the ratio


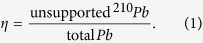


In recent decades levels of atmospheric lead pollution have decreased by an order of magnitude in many countries (e.g. the UK, see Fig. 3). The mean annual flux of ^210^Pb can be regarded as relatively constant. However, because of radioactive decay, the ^210^Pb/Pb ratio for material deposited at time *τ* will be a function of the time *t* of measurement. Denoting the initial ratio by


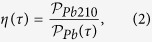


where 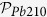
is the (constant) ^210^Pb flux and 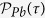 the Pb depositional flux at time *τ*, the ratio at a later time *t* will be


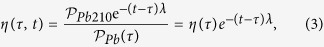


where *λ* is the ^210^Pb radioactive decay constant. Figure 3 also shows how the ratio of ^210^Pb to Pb deposited in the Richmond Park area (based on the monitoring data and decay corrected to 2012) has changed since 1970.

The ^210^Pb/Pb ratio in a given environmental sample collected at a given time *t* will depend on the relative significance of contributions from fallout in all previous years’ decay corrected to that time. If *F*_*i*_ denotes the fraction of the Pb concentration attributable to fallout deposited in the year *i* (*i* = 1, 2, … *n*) prior to time *t*, and *η*_*i*_ the ^210^Pb/Pb ratio in fallout deposited in year *i* decay corrected to time *t*, the ^210^Pb/Pb ratio in the sample will be


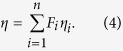


Figure 3 shows that values of this ratio may be expected to be relatively low in samples (e.g. soils) where the concentrations are dominated by older fallout or high amounts of minerogenic Pb. Much higher values may be expected in samples (e.g. mosses and some plant materials) where there has been a high uptake of freshly deposited Pb and ^210^Pb. In some dusts (e.g. particles generated from vehicles), the ratio tends to be zero as little unsupported ^210^Pb associated with them. Measurements of this ratio may therefore give a useful insight into the sources of Pb in environmental samples, and to the potential importance of remobilization of the large amounts of legacy Pb deposited over the years and presently stored in soils.

This method should have wide potential applications, especially in a time of climate change where the increasing fragility of catchment soils is likely to cause increased levels of soil erosion^17^. This could result in much higher rates of transport of legacy Pb from terrestrial catchments to lakes and rivers, counteracting current efforts to reduce contaminant levels in these water bodies. In view of this, monitoring Pb mobilisation in these environments will become increasingly important.

## Results and Discussion

### Impact of the ashing process on ^210^Pb and total Pb in plant samples

Since most plant samples have very low ^210^Pb activities, the samples need to be ashed to concentrate the ^210^Pb for measurement. Moss samples were ashed to investigate the impact of the ashing process on the ^210^Pb/Pb ratio (see Methods). The results of the measurements of unsupported ^210^Pb and total Pb in moss samples heated to different temperatures are shown in Fig. 4. Although the plot of ^210^Pb concentrations versus mass reduction factor (Fig. 4a) suggests increasing losses at higher temperatures, the ^210^Pb/Pb ratio (Fig. 4b) remains relatively constant, indicating that the ashing process can safely be used to concentrate ^210^Pb and Pb when determining this ratio in plant samples.

### Rainwater measurements

Table 1 shows the ^210^Pb activities, Pb concentrations and their ratios in rainwater samples collected between August and October 2012, and their mean values. The ^210^Pb activities are significantly higher than those in rainwater collected in Cumbria (UK)^18^, presumably reflecting the lower precipitation in London. Lead concentrations ranged from 1.67 mg m^−3^ to 2.1 mg m^−3^, with a mean value of 1.9 mg m^−3^. The mean value of the ^210^Pb/Pb ratio (*η*_d_) was 96 ± 9 Bq mg^−1^.

### Fallout Pb and ^210^Pb in soils

Figure 5 shows the distribution of unsupported ^210^Pb, Pb, and the ^210^Pb/Pb ratio in a soil core taken from a *Pteridium aquilinum* field at site A and sectioned at 2 cm intervals. Also shown are concentrations of the artificial fallout radionuclide ^137^Cs. Pb concentrations increase from around 40 mg kg^−1^ in the deeper layers below 13 cm to reach a peak value of 264 mg kg^−1^ in the 4–6 cm section before declining to around 50 mg kg^−1^ in the uppermost layer. ^137^Cs concentrations also have a peak value in the 4–6 cm section, suggesting that soils from this depth date from the mid-1960s. However, since soils do not normally contain a true sequential record this may not necessarily be the case. Unsupported ^210^Pb was above the level of detection only in soils above 8 cm. Concentrations peaked in the 2–4 cm section and showed a small decline in the uppermost layer, likely due to dilution by the less decomposed plants. The ^210^Pb/Pb ratio has its highest value (4.7 Bq mg^−1^) in the uppermost (0–2 cm) section though its value here is just 5% of that in the rainwater samples. Below this it falls rapidly with depth to a value of just 0.2 Bq mg^−1^ in soils between 4–8 cm, and is effectively zero in soils deeper than 8 cm. Results from other soil samples analysed in less detail are shown in Table 2. For the three park sites, significant levels of unsupported ^210^Pb were detected only in the uppermost 8 cm at site A, and uppermost 2 cm at sites B and C. Since at all three sites highest values of the ^210^Pb/Pb ratio were at least an order of magnitude lower than in rainwater it appears that even in near surface soils the Pb record is dominated by deposition prior to the year 2000 (Fig. 3). Soils from the garden site (D) appear to be well mixed, with relatively high Pb concentrations throughout the entire depth of the sample (40 cm).

### Uptake of Pb and ^210^Pb by plants

Table 3 gives the results of the Pb and ^210^Pb measurements on a range of vegetation samples from sites A–D. Most samples were ashed in order to improve the analytical accuracy, particularly for the radiometric measurements. Concentration factors due to ashing had an average value of 17, though in some samples were as high as 40. Where samples have been ashed the ^210^Pb activities and Pb concentrations are relative to the ashed weights. Also shown are estimates of the contribution of contemporary fallout to the Pb concentrations calculated using eq. (6) (see Methods).

The moss samples (*Pseudoscleropodium* and *Brachythecium*) all had similar unsupported ^210^Pb activities (mean value 77 Bq kg^−1^) and similar Pb concentrations (mean value 3.6 mg kg^−1^). Values of the ^210^Pb/Pb ratio (*η*) ranged from 19.1–24.5 Bq mg^−1^, and the calculated contribution of Pb from atmospheric deposition to total Pb from 18.1–25%. Shotyk *et al.*^19^ demonstrated complexity of Pb absorption by mosses, and revealed possible impacts of dusts and their particle sizes on Pb content in the mosses. Lead-210 in the atmosphere is derived exclusively from radioactive decay of ^222^Rn, and it tends to be attached to the surface of sub-micron aerosols. Lead contained by large particles in the deposition is less efficient than ^210^Pb to be uptaken by the plants. Therefore, actual Pb contribution from atmospheric deposition to the mosses might be less than calculated values. Because of their undeveloped root system, mosses are normally assumed to obtain most of their nutrient supply directly from atmospheric deposition and in consequence have been widely used to monitor atmospheric trace metal pollution^5^,^20^. The above results suggest that the Richmond Park mosses derive much of their Pb from the soils or the dusts, significantly reducing their ^210^Pb/Pb ratio. This might be due to the relatively low rainfall in London (average 650 mm per annum^21^), resulting in insufficient nutrients and water for moss growth^22^,^23^, and dust impacts as the ground area where the mosses were collected was covered in around 40–90% of the surface by living vegetation. The ^210^Pb/Pb ratios in the Richmond mosses are about one tenth of those in the moss samples collected in 2013 from Grampian Mountains and Galloway in Scotland, and the lake Distract in Northern England (unpublished data), where the annual rainfall is 2 or 3 times higher than that in London, and ground vegetation overages are also higher. These results reveal a possible limitation in the use of mosses to monitor air pollution, particularly at low rainfall sites. *Arrhenatherum elatius* samples collected from dry soils in sites A and B also have relatively high ^210^Pb/Pb ratios, similar to those in mosses, while *Juncus effusus* samples collected from puddles have relatively low ratios that might have been affected by the water on the ground.

*Pteridium aquilinum* (Bracken) is an annual plant with stems and leaves. The leaves have relatively high ^210^Pb/Pb ratios, suggesting significant inputs directly from atmospheric deposition. In contrast, the stems, which grow very quickly in spring and take their nutrient from the soils, have very low values. *Pteridium aquilinum*, *Rubus fruticosus*, *Pseudosasa* and *Hedera helix* also have significant amounts of unsupported ^210^Pb in the leaves but negligible amounts in their branches. This suggests that here too the leaves absorb a significant proportion of their Pb directly from the atmosphere, but uptake by other parts of the plant is mainly from the soils. Although *R. ponticum* leaves and branches both contain relatively high Pb concentrations, the low ^210^Pb/Pb ratios suggest that this tree species takes nutrients and water mainly from the ground via its roots. In C*astanea sativa* and *Quercus robur*, the leaves and branches have similar ^210^Pb/Pb ratios. As the trees have been growing for many years, radioactive decay will have reduced the ^210^Pb/Pb ratio for inputs derived both from direct atmospheric absorption and uptake from soils. Although in this case it is difficult to assess the atmospheric contribution, the similar ratios in the leaves and the branches would imply that the nutrient exchange or mixing between the leaves and branches is relatively fast.

The contributions from atmospheric deposition to Pb concentrations in the leaves of some plants such as *Rubus fruticosus*, *Pteridium aquilinum*, *Pseudosasa* and *Thuja occidentalis* are similar to or even higher than those in mosses. The leaves of these plants may thus have potential use for monitoring air pollution (e.g.^8^,^16^,^24^,^25^). Low atmospheric contributions of Pb in the leaves of *Quercus robur*, *Fagus sylvatica*, *R. ponticum* and C*astanea sativa* trees might suggest that these trees take nutrients and water supply mainly from the ground via their roots, or that the composition in the leaves is significantly influenced by that in their tree trunks. Consequently, they may not be suitable for air pollution monitoring.

Pollutant fate and transport in the environment are becoming increasingly important in both regulatory and scientific areas. With a predicated increase in extreme weather events, remobilisation of Pb stored in the environment will be enhanced. There is an urgent need to monitor the Pb remobilisation to assess secondary pollution. Signatures of stable Pb isotopes can be used to identify possible anthropogenic and natural sources (e.g.^9^,^19^,^26^), while as shown in this first example, the ^210^Pb approach can be used to indicate and even quantify Pb contribution from atmospheric deposition, track Pb sources into plants and its transportation within the plants. Stable Pb isotopes have been widely used in environmental research, the ^210^Pb method also has potential to be used extensively in different environmental settings, not only revealing Pb sources, but also monitoring Pb transportation.

## Materials and Methods

### Sampling

**Figure 1:**
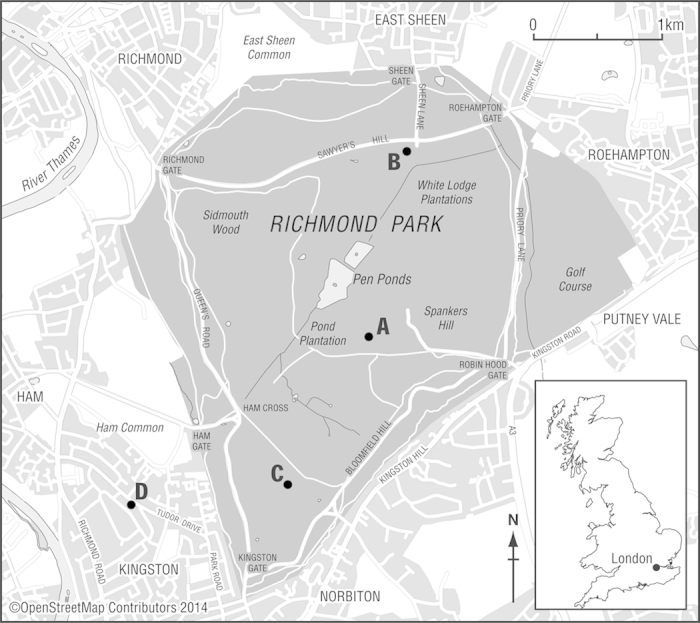
Map of Richmond Park and locations of sampling sites. Sites A, B and C are in the Park, site D is in a residential garden where rainwater samples were collected. The map is based on OpenStreetMap Contributors open data, licensed under the Open Data Commons Open Database License (ODbL) by the OpenStreetMap Foundation (OSMF), 2014 (for further copyright information visit www.openstreetmap.org/copyright). Created by the Drawing Office, UCL Department of Geography, using Adobe CS5.1.

**Figure 2:**
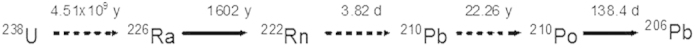
^238^U decay series, showing the principal radionuclides concerned with the production of ^210^Pb and their radioactive half-lives. (Dashed arrows indicate decay through one or more intermediate radionuclides).

**Figure 3:**
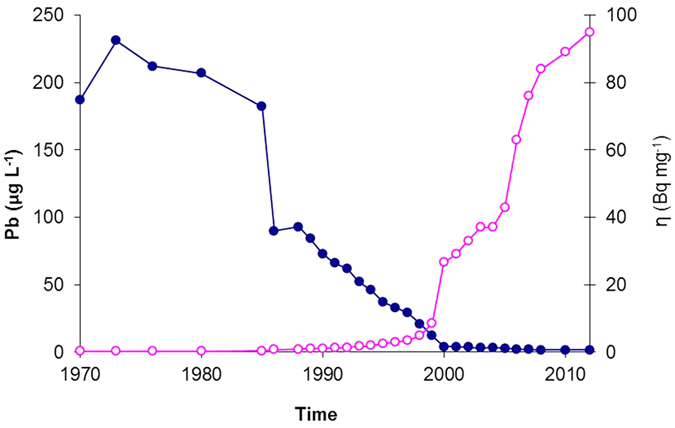
Temporal trends in total Pb concentrations in atmospheric deposition (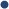) based on monitoring data and UK emission trends^30^ and calculated levels of the decay corrected ^210^Pb/Pb ratio *η* (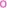) through time at Richmond Park area, London.

Soil and plant samples were collected from three sites across Richmond Park from September to October 2012 (A, B and C in Fig. 1). Soil samples were dug out using stainless steel knives, the depth of sampling (see Table 2) being mainly determined by changes in the soil organic content and colour. A soil core was taken from a *Pteridium aquilinum* field at site A and sectioned at 2 cm intervals. Plant samples were collected using gloved hands, and tree branches cut using a stainless steel tree branch cutter. Plant samples were carefully washed prior to analysis using deionised distilled water. For security reasons, bulk deposition samples were collected at a nearby residential garden (around 600 m from the Park) during August to October 2012, using a rigorously cleaned polypropylene collector placed 1.5 m above ground. The collections were checked every 12 h. Those contaminated by birds, flies or other particles such as resuspended soils or dusts were discarded, and the collector extensively rinsed with deionised distilled water before replacing. Uncontaminated samples were transferred to closed Teflon bottles and stored in a refrigerator at around 4 °C. Samples collected during the course of each calendar month were combined to form single monthly samples. Each was divided into two parts, 50 ml was acidified to 1% by HNO_3_ for Pb analysis, and the rest (>0.5 L) used for ^210^Pb analysis. Soil and plant samples were also collected from this site.

### Total Pb analysis

Soils samples were analysed by using a Spectro XLAB2000 X-ray fluorescence (XRF) spectrometer. Vegetation samples (0.2 g) were digested for 2 hr at 100 °C using 6 mL concentrated HNO_3_, and then diluted to 50 mL with deionised distilled water. The digested solutions and rainwater samples were analysed using ICP-MS. Standard soil and vegetation reference materials were digested and analysed, and had recovery values in the range of 95–102% of the certified values. Standard errors for the water sample analyses were estimated to be less than 10%. A conservative figure of 10% has been used in all calculation.

### Lead-210 analysis

**Figure 4:**
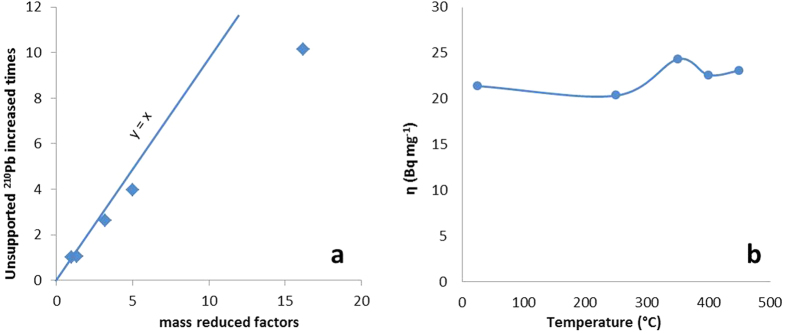
Relationships of unsupported ^210^Pb activities, Pb concentrations with moss mass reduction during sample ashing process at different temperatures. (**a**) relationship of increase in unsupported ^210^Pb activity with mass reduction. (**b**) ratios of unsupported ^210^Pb activity to Pb concentration (*η*) are relatively constant in moss when temperature increases.

**Figure 5:**
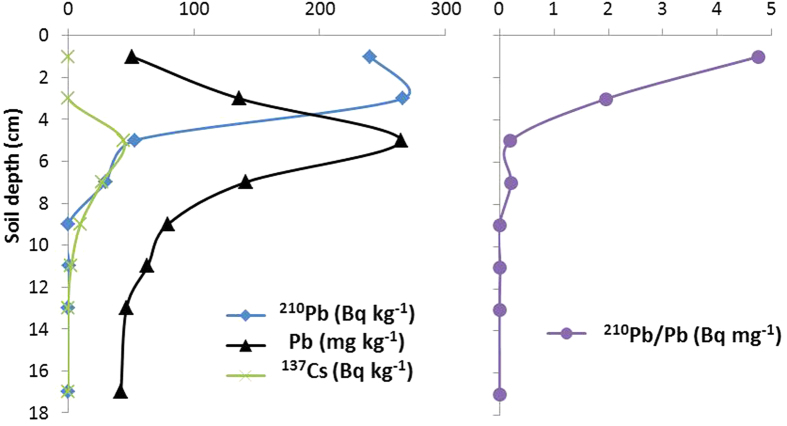
Distributions of unsupported ^210^Pb, Pb and ^137^Cs, and ratios of ^210^Pb activities to Pb concentrations in a soil profile taken from Richmond Park. The soils in this profile taken from a *Pteridium aquilinum* field in Site A (Fig. 1) are peaty. A ^137^Cs peak in the 4–6 cm suggests that the soils in this depth were formed around the mid-1960s. The Pb concentration profile might follow the pollution history in the area. The maximum level of ^210^Pb below the soil surface is possibly due to decomposition of the dead plants. ^210^Pb/Pb ratios in the surface soils are considerably lower than that in the atmospheric deposition (cf. Table 1), and reduced quickly to zero with depth.

Radionuclides in rainwater samples were removed from solutions by co-precipitation with manganese dioxide^27^, separated from the supernatant liquid by filtration through a 0.45 μm membrane filter and dried. Plant samples with low ^210^Pb activities, were ashed at 420 °C to concentrate the ^210^Pb. Moss samples from site A were also heated separately to 250 °C, 350 °C, 400 °C and 450 °C to investigate the impact of the ashing process on the ^210^Pb/Pb ratio (see Fig. 4). All the samples were analysed for ^210^Pb, ^226^Ra and ^137^Cs by direct gamma assay in the Environmental Radiometric Facility at University College London, using ORTEC HPGe GWL series well-type coaxial low background intrinsic germanium detector. Lead-210 was determined via its gamma emissions at 46.5 keV, and ^226^Ra by the 295 keV and 352 keV gamma rays emitted by its daughter isotope ^214^Pb following 3 weeks storage in sealed containers to allow radioactive equilibration. Caesium-137 and ^241^Am were measured by their emissions at 662 keV and 59.5 keV^28^. The absolute efficiencies of the detector were determined using calibrated sources and sediment samples of known activity. Corrections were made for the effect of self absorption of low energy gamma rays within the sample^29^. In each sample unsupported ^210^Pb activity, the fallout component, was calculated by subtracting supported ^210^Pb (i.e. ^226^Ra) activity from total ^210^Pb activity. Standard errors (1*σ*) have been calculated from the counting statistics.

### Calculation of Pb contribution to the plants

Sources of Pb (and unsupported ^210^Pb) in plants can include both contemporary atmospheric fallout, and historic fallout stored in the soils that include plant absorption through roots in the soils and through leaves from resuspended dusts. In remote area, the dusts are mainly composited by resuspended surface soils. If *η*_*d*_ is the measured ^210^Pb/Pb ratio in contemporary deposition, *η*_*s*_ is the measured ^210^Pb/Pb ratio in soils feeding the plants, *F*_*d*_ is the fraction of Pb in plants arising from contemporary atmospheric fallout, and noting that the soil fraction (including soils through roots and dusts through leaves) will be 1 − *F*_*d*_, then the (measurable) plant ^210^Pb/Pb ratio (c.f. eq. (4)) will be:


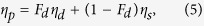


Hence, the fraction arising from contemporary atmospheric fallout will be:


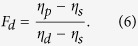


Effect of road dust on plants 20 m away from the road is normally low and impact on Pb concentration is small^26^), and unsupported ^210^Pb in vehicle generated dust tends to zero. Our sampling locations in the park are far away from the road. Therefore, we put vehicle transport dusts into soil dust category when calculating Pb contribution from atmospheric deposition to the plants.

It is difficult to work out dust contribution though leaves and soil contribution through roots separately by using this ^210^Pb method. Nevertheless, as there is a considerable difference between ratios in atmospheric deposition and others (i.e. soils or other dusts), Pb contribution to plant by atmospheric deposition can be calculated or estimated.

Lead and ^210^Pb concentrations in soils vary both with location and soil depth. The mean 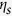 of Pb in the soils of different depths that contributes to the connected root is:


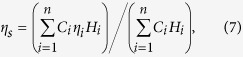


where *C*_*i*_ and *η*_*i*_ is Pb concentration and its ^210^Pb to Pb ratio, respectively, in every soil layer *i* that connected with the root, *H*_*i*_, is thickness of the soil layer *i*. In practice, the values used for plant sample calculations were those for that with similar type soils containing the root ball. As mosses rarely have roots, the value of *η*_*s*_ used for calculating soil Pb contribution to the mosses is the *η*_*s*_ in the surface soils underneath. However, unsupported ^210^Pb concentrations are usually significantly above zero only in the uppermost 10 cm soils (and often just the uppermost 5 cm), and tree roots usually extend to much greater depths, the value of *η*_*s*_ in these cases can normally be assumed to be zero, particularly where it has a low value in the surface soils. Since fallout in earlier years may be stored in tree trunks growing from those years, concentrations in two or three year-old tree branches were analysed in order to determine the sources influencing Pb absorption in leaves. If ^210^Pb to Pb ratio in plant organs tends to be zero, it suggests that contribution of unsupported ^210^Pb from soils is little.

## Additional Information

**How to cite this article**: Yang, H. and Appleby, P. G. Use of lead-210 as a novel tracer for lead (Pb) sources in plants. *Sci. Rep.* **6**, 21707; doi: 10.1038/srep21707 (2016).

## Figures and Tables

**Table 1:** ^210^Pb activities, Pb concentrations and their ratios in rainwater samples.

Collection period	^210^Pb		Pb		^210^Pb/Pb (*η*_*d*_)	
	Bq kL^−1^	±	mg kL^−1^	±	Bq mg^−1^	±
August 2012	148	22	2.1	0.21	70	13
September 2012	180	16	1.67	0.17	108	15
October 2012	215	32	1.93	0.19	111	20
*Mean values*	*181*	*14*	*1.9*	*0.11*	*96*	*9*

**Table 2:** Unsupported ^210^Pb, Pb and ^137^Cs and ratios of ^210^Pb activities to Pb concentrations in soil samples collected from Richmond Park.

Site	Depth	Unsupported ^210^Pb		Pb		^210^Pb/Pb (*η*_*s*_)		^137^Cs	
	cm	Bq kg^−1^	±	mg kg^−1^	±	Bq mg^-1^	±	Bq kg^−1^	±
A	0–1	61.8	11.6	81.2	8.1	0.76	0.16	9.05	2.29
	0–5	73.3	11.8	123.9	12.4	0.59	0.11	31	1.95
	5–10	0		45.8	4.6	0		1.67	0.5
	10–20	0		38.3	3.8	0		0	
B	0–2	29.91	3.7	72.2	7.2	0.41	0.07	9.63	0.58
	2–7	0		63.4	6.3	0		7.07	0.48
C	0–1	64.6	19.9	28.8	2.9	2.24	0.73	0	
	0–2	139.1	21.3	78.7	7.9	1.77	0.32	7.59	2.65
	2–7	0		79.6	8.0	0		4.94	1.2
	7–17	0		42.1	4.2	0		0	
D	0–10	4.6	5.8	138	13.8	0.03	0.04	0	
	10–20	0		96	9.6	0		0	
	20–40	0		156	15.6	0		0	

**Table 3:**
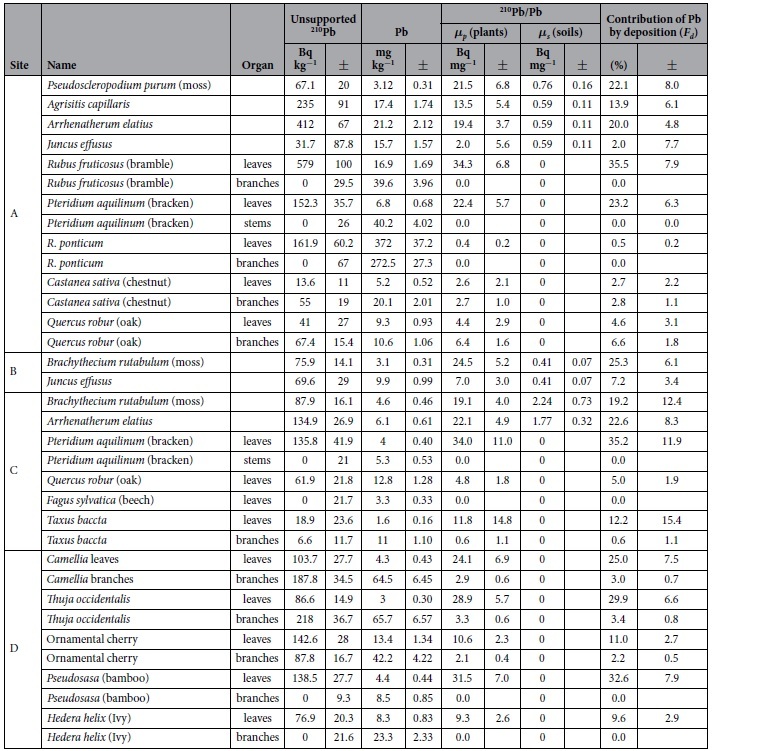
Unsupported ^210^Pb activities, Pb concentrations and their ratios in vegetation samples, *η*_*s*_ in soils used for calculating Pb uptaken by plant root, and contributions of Pb to the vegetation by atmospheric deposition.
